# Intraspecific variation in feeding strategies of Galapagos sea lions: A case of trophic specialization

**DOI:** 10.1371/journal.pone.0185165

**Published:** 2017-10-23

**Authors:** Diego Páez-Rosas, Stella Villegas-Amtmann, Daniel Costa

**Affiliations:** 1 Universidad San Francisco de Quito (USFQ) and Galapagos Science Center, Galápagos, Ecuador; 2 Dirección Parque Nacional Galápagos, Unidad Técnica Operativa San Cristóbal, Galápagos, Ecuador; 3 Department of Ecology and Evolutionary Biology, University of California at Santa Cruz, Santa Cruz, United States of America; Phillip Island Nature Parks, AUSTRALIA

## Abstract

The trophic behavior of marine predators varies according to the level of competition to which they are exposed. In general, populations that inhabit lower productivity systems face a strong intraspecific competition, which contributes to the development of different foraging strategies to maximize nutritional efficiency. Given the high trophic flexibility of *Zalophus wollebaeki*, this species is considered appropriate for the analysis of such behavior. Furthermore, this trophic flexibility has allowed them to persist in a seemingly marginal ecosystem. In this study, we used a comparative analysis of variables (diet and dive behavior) related to *Z*. *wollebaeki* trophic niche plasticity to better understand their foraging ecology, using techniques such scat analysis, satellite telemetry and complementarily an isotopic analysis. Scat analysis revealed intra-population variation in their diet, represented by prey from different environments (epipelagic and benthic). These results are supported by the animals’ locations at sea and diving profiles. Global Positioning System (GPS) and time-depth recorder (TDR) records showed the existence of two groups, with differing feeding areas and diving behavior. Also the δ^15^N values showed differences in the trophic level at which the species fed. These results constitute a relevant finding in the evolutionary behavior of the species, showing that *Z*. *wollebaeki* has developed a high degree of foraging flexibility, thus increasing its survival rate in an ecosystem that is highly demanding in terms of resource availability.

## Introduction

Top marine predators show high heterogeneity in their foraging behavior as a result of variation in habitat type, marine productivity, competition degree, age and size [1,2]. Therefore, a single population can exhibit groups of individuals with different diets that can comprise prey from different environments [3,4]. For decades, all individuals within a population or species were believed to occupy the same trophic niche [5,6]. However, diversification of feeding habits at inter- and intra-population levels is frequently observed in nature [7,8]. In several species of marine predators, this behavior is thought to be an ecological and evolutionary adaptation, influencing the population dynamics of these species [9,10]. Moreover, this diversification offers an advantage when competing for resources, by reducing trophic competition and enabling coexistence in areas with limited resources [11,12].

Trophic studies based on the 'optimal foraging theory' of different marine predators predict that individuals in a population should only feed on the most abundant and energy-rich resources to optimize energy intake per unit time [13]. Therefore, if a population has access to a higher diversity of prey, the individuals could diversify their diet to reduce competition [14,15]. Consequently, marine predators can exhibit variability in their prey search behavior, based on their physiological capacity, providing the species with a wide range of alternatives when choosing their prey [16,17]. Thus, individuals from the same population can feed on different prey, resulting in diets that vary based on the individual’s feeding niche [7,18]. The diversification of feeding strategies is usually promoted by the availability of open niches, as a consequence of reduced inter- and intraspecific competition or high heterogeneity of foraging areas [14]. Hence, top predators that inhabit areas with high prey diversity, exhibit some degree of trophic specialization [19,20].

Pinnipeds are high-trophic-level consumers that forage on a wide range of prey from different environments. Such environments may vary both geographically and over time [21,22]. However, this variability is more evident in tropical species that encounter greater uncertainty of marine productivity. This uncertainty translates into changes in the abundance and distribution of their main prey [23,24]. Otariids of the Galapagos Islands exhibit variations in diet composition that have been related to competition levels and their capacity to recover under nutritional stress [25,26]. These species inhabit a region where the levels of marine productivity are strongly influenced by ocean currents and by a pattern of upwelling that make this area a biodiversity hotspot where several species of marine predators congregate [27,28]. Such factors contributed to the sporadic colonization of the islands and led to the evolution and presence of the divergent species that are currently found in this region [29,30].

The Galapagos sea lion (*Zalophus wollebaeki*) represents an interesting case study because it is a highly philopatric species with great mobility to exploit different environments during its foraging trips [31,32]. Diet studies of *Z*. *wollebaeki* have identified some degree of specialization given that a high percentage of its diet, is composed of a few number of prey species [3,24]. Diving behavior studies on the Galapagos sea lion population of Caamaño islet and Fernandina Island show that they use at least three foraging strategies which depend on the dive depth (epipelagic, mesopelagic and benthic). These suggest the existence of different diets in individuals from the same population [26,31].

The combination of traditional techniques, such as scats analysis, along with more complex techniques, such as satellite telemetry, provides a better interpretation of the trophic ecology of a given species. Scat analysis provides taxonomic information of the prey consumed by recovering and identifying species structures, such as fish otoliths and cephalopod beaks [33]. Global Positioning System (GPS) transmitters and time-depth recorders (TDR) are extensively used in foraging behavior studies of marine predators, as they generate information on their horizontal and vertical temporary displacement [34,35]. The foraging patterns of marine vertebrates can be classified by their foraging habitat, such as pelagic when they feed in the water column or benthic- near the bottom of the continental shelf [31,36]. This information can be complemented by other techniques such as the stable isotopes analysis of nitrogen and carbon. This technique allows inferences to be made about the trophic level and the preferential habitat occupied by the predators, based on the physical, chemical and biological factors of the environment [37,38]. In the case of δ^15^N, bioaccumulation occurs in the content of the ^15^N between a predator and its prey, causing isotopic enrichment between trophic levels [39]. The variation of δ^13^C values in aquatic predators depends on the habitat type of its prey: coastal/oceanic or pelagic/benthic [37,40].

In this study, we applied all these techniques used in trophic ecology studies, to investigate in depth the trophic behavior of *Z*. *wollebaeki*. We aimed to identify the possible existence of diverse foraging strategies in the San Cristobal population, possibly influenced by intraspecific competition and the oceanographic characteristics of the region.

## Methods

### Ethics statement

This research was performed as part of the *Z*. *wollebaeki* population monitoring program conducted by the Galapagos National Park, under the research permits PC-15-09, PC-60-10, PC-46-15 and was financial supported by the University of California at Santa Cruz. This research was carried out following the protocols of ethics and animal handling approved by the Galapagos National Park and University of California.

### Study area and sample collection

This study was conducted at the “El Malecón” rookery in San Cristóbal Island (0°54´8.1´´S, 89°36´44.1´´W), during Nov-Dec 2009, at the end of the breeding season. Currently, this rookery is the largest of the Archipelago, with an average of 630 individuals, of which 55% are adult females, 31% are juveniles, and 14% are adult males [41]. In order to monitor the feeding habits of this population, we focused on adult females, as they represent the most important sex/age class category.

### Scat analysis

We collected 60 scat samples at the “El Malecón” rookery in areas with adult female predominance in order to minimize the presence of other sex/age classes in the analysis. We collected several scats only once per day in different areas of the rookery, assuming that each scat was from a single individual. To determine if the sample size adequately represented the trophic spectrum of the population, we utilized the model applied to Galapagos sea lions by Páez-Rosas & Aurioles-Gamboa 2010. This model calculates the cumulative average and standard deviation of a group of diversity curves obtained from random permutations of the original data.

Subsequently, to determine the diet composition, we used the index of importance (IIMPi) value of main prey proposed by García-Rodríguez & De La Cruz-Agüero 2011 [42], which estimates the importance of prey species in each sample unit, including the probability of finding these species in the total sample. This index produces results that range from zero to one, but for subsequent analyses, the values were converted into percentages (IIMPi x 100). We then selected all the prey with IIMPi higher than 5% and performed a principal component analysis (PCA) to identify the presence of one or more specific diets within this rookery, following the criteria proposed by Páez-Rosas & Aurioles-Gamboa 2014.

### Satellite telemetry and TDR deployment

To analyze the spatial movements, habitat characteristics, and diving behavior of *Z*. *wollebaeki*, we captured 10 random nursing females at the “El Malecón” rookery in November 2009. Animals were captured using a cone-shaped net with an opening at the end, from which animals were able to protrude their nose and breathe normally while being manually restrained and instrumented. This work was carried out in conjunction with a study that examined the foraging energetics of this population [43].

We instrumented sea lions with nine Mk10-AF tags (Wildlife Computers, Richmond, WA, USA) and one Sirtrack tag (Sirtrack Ltd., Havelock North, New Zealand), coupled with a MK9 TDR (Wildlife Computers, Richmond, WA, USA) to determine the diving and movement patterns. Additionally, all animals were equipped with radio transmitters (VHF; Sirtrack, Havelock North, New Zealand) to facilitate their recapture on land after the animals had returned from their foraging trips (approximately two weeks). The instruments were deployed on the dorsal region at shoulder height. Instruments were mounted on a Neoprene base and a mesh attached with Loctite epoxy on to the fur of the animal. The gross weight of the instruments was approximately 293 g (0.4% of the mass of the animal), and most likely did not affect the normal behavior of the individuals [31,43]. After instrument recovery, the mesh and epoxy residues on the coat of the animal were lost during the annual molt of the animals.

To determine the foraging areas of San Cristobal females, GPS positions were decoded with a distributed array processor (DAP) (Wildlife Computers, Richmond, WA, USA). Data were then filtered with a routine (IKNOS toolbox, Tremblay, unpublished) written in MATLAB (MathWorks Inc., Natick, MA, USA), which uses several criteria to eliminate improbable data [44] S1 File. The filtered data were then mapped using ArcGIS 10.1 (ESRI Inc., Redlands, CA, USA). Diving data were analyzed using a diving analysis program in MATLAB (IKNOS toolbox, Tremblay, unpublished) that allows dives to be identified on the basis of minimum depth and duration S2 File. The minimum depth considered as a dive was 5 m, and the minimum duration was 12 s.

The spatial segregation of the species was estimated on the basis of the GPS position at each foraging trip and the previously georeferenced feeding areas. In order to identify the main foraging areas of the population, where most of the dives were concentrated, we used kernel density estimation (with a margin of 2 km) using a routine in ArcGIS 10.1. Furthermore, dives were analyzed and classified on the basis of their depth distribution in two categories: shallow (< 100 m) and deep (> 100 m). To identify diverse foraging strategies based on diving behavior; we performed a cluster analysis, using the Euclidian distance and the index of similarity of the nearest neighbor to measure the distance between groups.

### Stable isotope analysis (Complementary technique)

We collected hair samples from all the females instrumented (n = 10) to analyze and compare the differences in diet and foraging behavior, based on the measurement of nitrogen (δ^15^N) and carbon (δ^13^C) isotope ratios S3 File. This information complemented our results and allowed us to make inferences about the feeding patterns of the females from this rookery over time, as the isotope renewal rate in this tissue is approximately 3 months [3,37].

All samples were dried at 80°C for 12 hours and lipids removed according to the microwave-assisted extraction protocol [3,24]. The δ^13^C and δ^15^N isotope ratios were measured in a continuous-flow isotope-ratio-monitoring mass spectrometer (20–20 PDZ Europe) at the UC Davis Stable Isotope Facility. Results are expressed in parts per thousand (‰) according to the following equation: δ^13^C or δ^15^N = 1000[(R_sample_/R_standard_)-1], where R_sample_ and R_standard_ are the ratios of ^13^C/^12^C or ^15^N/^14^N for the sample and the standard, respectively. Normality and homoscedasticity of the isotopic data were analyzed by means of the Shapiro-Wilk and Levene tests, respectively. We performed Student’s t-tests to detect significant differences in the δ^13^C and δ^15^N values. Significance level was set at p = 0.05. All tests were performed using statistical software packages (STATSOFT Inc., Tulsa, OK, USA).

Finally, we calculated the trophic level of *Z*. *wollebaeki* using the isotopic signatures of the predator and the primary producer according to the algorithm proposed by Post 2002: TL = λ + [(δ^15^N_p_ − δ^15^N_pc_)/Δ_n_], where: δ^15^N_p_ and δ^15^N_sc_ are the respective isotopic signatures of the predator and primary consumer, λ is the trophic position of the primary consumer, and Δ_n_ is the value of the mean fractionation of δ^15^N between the different links of the trophic web of the predator. For this analysis, we used the isotopic signal of phytoplankton (δ^15^N_pc_) and the fractionation value adjusted to food web of *Z*. *wollebaeki* (Δ_n_) proposed by Páez-Rosas et al. 2012.

## Results

### Scat analysis

**Fig 1:**
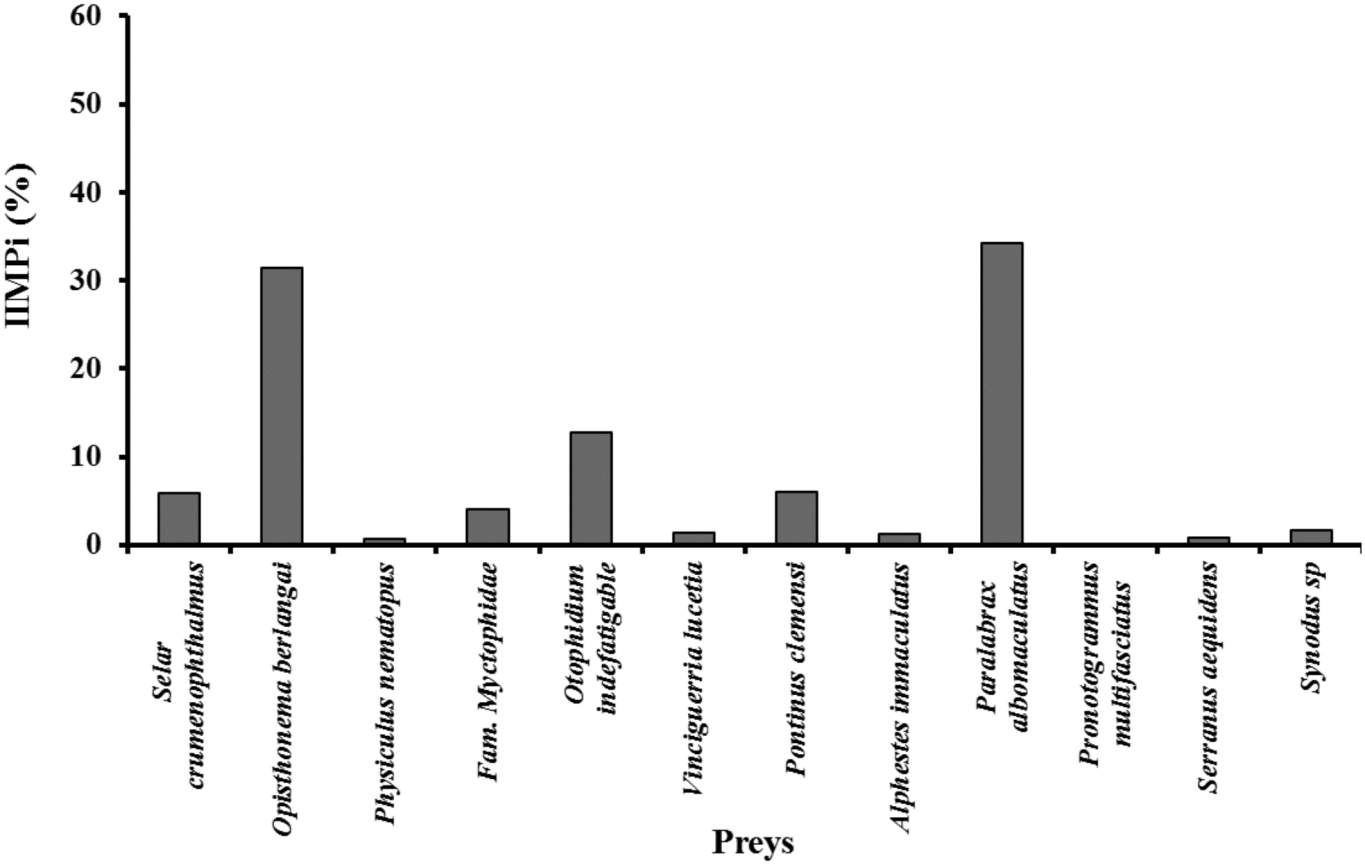
Importance index (IIMPi) values of Galapagos sea lions main prey species (≥ 5%) from scats collected at El Malecón rookery.

The diversity model used to determine the optimum sample size for the scat analysis was standardized at 46 scats, confirming that our sample size selected a priori was appropriate to represent the diet of *Z*. *wollebaeki* in this rookery. The diet spectrum of the species was relatively narrow and comprised 12 items (all fish). Of these, 11 prey (92%) were identified to the level of species, and one to the level of family (8%) (Table 1). Only prey with an IIPMi value greater than 5% were considered important in the diet. On the basis of this criterion, six main prey types representative of different depths and trophic levels (Table 1), were detected: “Camotillo” (*Paralabrax albomaculatus*) (34.22%), “Galapagos sardine” (*Opisthonema berlangai*) (31.39%), “Congriperla” (*Otophidium indefatigable*) (12.69%), “Brujo” (*Pontinus clemensi*) (6.01%), “Ojón” (*Selar crumenophthalmus*) (5.84%) and fish of family Myctophidae (4.01%) (Fig 1).

**Table 1:** 

N°	Family	Species	# Scats	# Otolits	IIMPi	* Trophic level		Habitat
1	Carangidae	*Selar crumenophthalmus*	11	18	5.84	Planktivore	3.0	Epi-pelagic
2	Clupeidae	*Opisthonema berlangai*	20	32	31.39	Planktivore	2.9	Epi-pelagic
3	Moridae	*Physiculus nematopus*	2	2	1.01	Carnivorous	3.4	Benthic
4	Myctophidae		10	20	4.01	Planktivore	3.0	Meso-pelagic
5	Ophidiidae	*Otophidium indefatigable*	15	18	12.69	Carnivorous	3.5	Demersal
6	Phosichthyidae	*Vinciguerria lucetia*	4	4	1.74	Planktivore	3.0	Meso-pelagic
7	Scorpaenidae	*Pontinus clemensi*	10	22	6.01	Carnivorous	3.5	Demersal
8	Serranidae	*Alphestes immaculatus*	2	2	1.01	Carnivorous	3.5	Reef
9	Serranidae	*Paralabrax albomaculatus*	22	34	32.22	Carnivorous	3.8	Reef
10	Serranidae	*Pronotogrammus multifasciatus*	2	4	1.33	Carnivorous	3.1	Reef
11	Serranidae	*Serranus aequidens*	4	4	1.74	Carnivorous	4.0	Reef
12	Synodontidae	*Synodus lacertinus*	2	3	1.01	Carnivorous	4.2	Benthic

* The trophic level of preys was obtained from Fish Base (www.fishbase.org).

**Fig 2:**
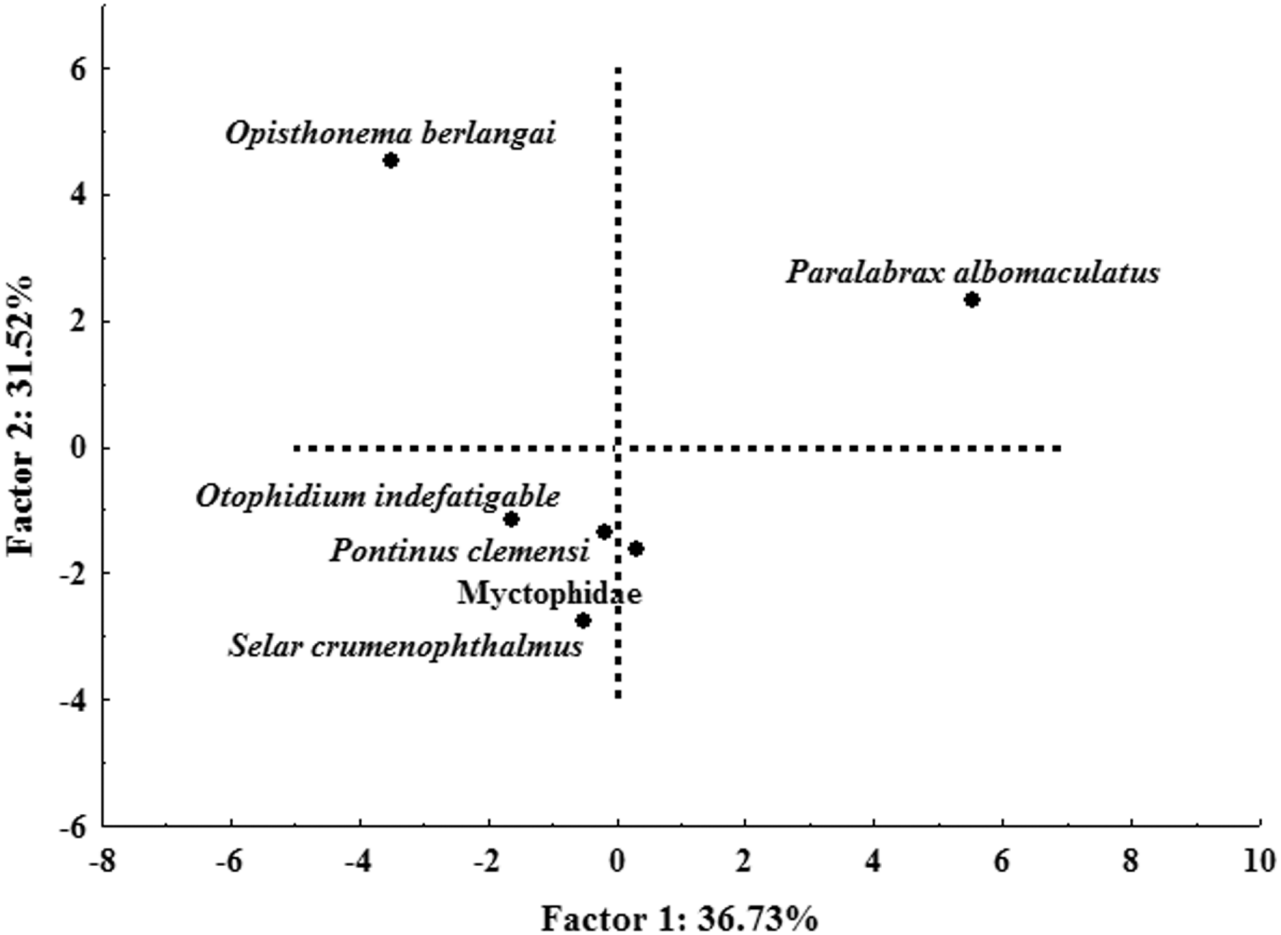
Principal component analysis (PCA) of the importance index (IIMPi) values of Galapagos sea lions’ prey, from El Malecón rookery, San Cristobal Island.

The principal component analysis (PCA) identified the presence of five principal components. PC1 and PC2 accounted for 68.25% of the total data variance. PC1 showed a positive correlation with scats in which the principal prey was *P*. *albomaculatus* and a negative correlation with scats that contained other types of prey. PC2 presented a positive correlation with scats in which the principal prey was the *O*. *berlangai* and a negative correlation with the remaining scats. Based on the scat analysis, we identified at least three different diets within the same population (Fig 2).

### Satellite telemetry and TDR analysis

Female satellite tracking duration ranged between 5–13 days (mean 7.44 ± 2.87 d), during which an average of 23.2 geographical positions were registered (range = 16–30) for each of the instrumented females. Females exhibited a mean of 2.6 ± 1.9 foraging trips, and spent an average of 61.3 ± 9.3% of their time at sea. The mean maximum distance covered during these foraging trips, relative to the rookery location, was 46.3 ± 16.1 km, with duration of 44.1 ± 18.6 h at sea and on land periods between 7.18–48.33 h (Table 2). The foraging trips occurred during the day and night, with no defined pattern.

**Table 2:** 

ID	Foraging location	Foraging trip					Diving behaviour		
		# Foraging trips	Mean trip duration (h)	* Max distance traveled (km)	# Days recorded	Max dive depth (m)	Mean dive depth (m)	Mean dive duration (min)	# Total dives
SCF01	North/West	2	23.8 ± 5.2	38.7	3.7	N/A	N/A	N/A	N/A
SCF03	North	1	84.3 ± 0	55.1	5.4	548	249.2 ± 203.3	5.8 ± 3.9	375
SCF06	North	1	63.1 ± 0	50.9	4.4	520	119.8 ± 177.5	3.3 ± 3.4	476
SCF07	North	1	55.4 ± 0	49.6	4.2	533	154.8 ± 199.5	3.8 ± 3.8	384
SCF08	North	4	30.9 ± 27.5	51.1	7.6	571	170.9 ± 191.6	4.6 ± 3.6	652
SCF02	West	4	43.4 ± 5.3	57.3	9.6	430	147.1 ± 134.1	4.6 ± 2.7	954
SCF04	West	2	30.8 ± 12.1	23.4	5.4	369	130.6 ± 108.4	4.3 ± 2.5	434
SCF05	West	7	30.6 ± 19.1	70.1	12.8	448	133.3 ± 107.6	4.3 ± 2.5	1043
SCF09	West	2	35.7 ± 0.7	20.3	4.4	330	126.1 ± 94.6	4.3 ± 1.9	523
SCF10	North	2	44.8 ± 2.7	46.3	6.9	517	145.6 ± 168.6	4.4 ± 3.4	527

* The maximum distance traveled is calculated in relation to the breeding rookery. The female SCF01 not dive data, it is unknown where directs their foraging trips.

**Fig 3:**
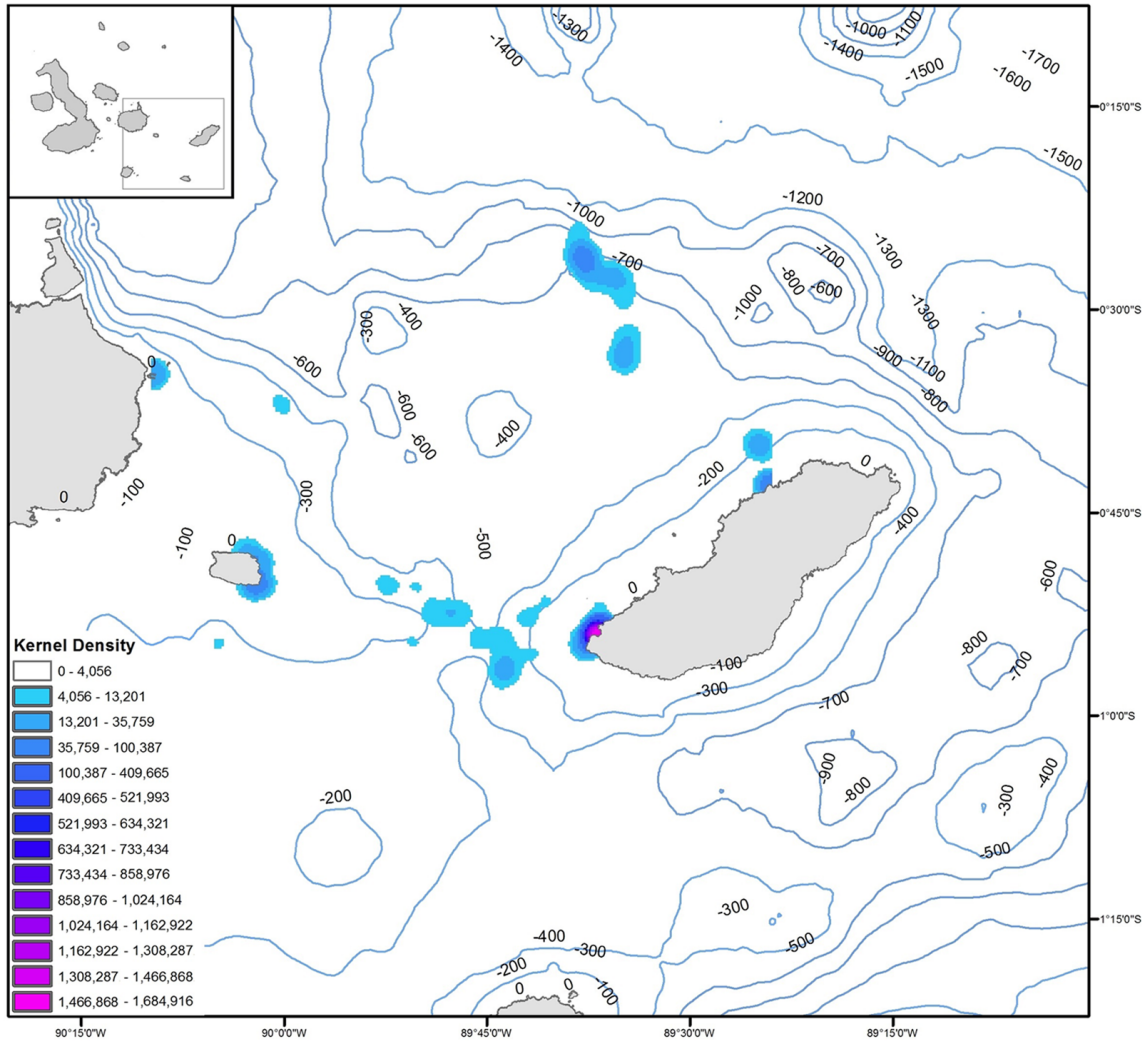
Foraging range of Galapagos sea lion females from El Malecón rookery, based on 95% kernel density contours. Additional contour lines indicate bathymetry at 100 m intervals.

The kernel density analysis revealed a spatial segregation of foraging areas. The highest concentration of diving sites was observed north and west of the island (Fig 3). Most females foraged in both continuous areas that were separated into two different nuclei, at a distance of approximately 55 km from each other (Fig 3). A third nucleus was observed around the rookery, which was used by the two groups of females.

Given that all females exclusively utilized one foraging area (either north of west of the Island); we compared the diving behavior or these two groups. The female SCF01 was excluded from the analysis since we could not obtain the dive data of this individual. We found significant differences in the diving behavior of the individuals occupying these foraging areas. When considering their deep dives (>100 m), females with foraging trips to the north of the island (n = 5) exhibited greater diving depth ability (ANOVA, P = 0.001, 413.8 ± 12.7 vs. 235 ± 29.5 m) and apnea (ANOVA, P = 0.001, 7.1 ± 0.2 vs. 6.5 ± 0.5 min) compared to females that foraged to the west (n = 4). Females foraging to the west exhibited a greater number of dives (ANOVA, P = 0.001, 738.5 ± 304.6 vs. 471.3 ± 129.1) and spent more time at the bottom of a dive (ANOVA, P <0.001, 1.4 ± 0.1 vs. 0.9 ± 0.1 min) compared to females that foraged north (Table 2).

**Fig 4:**
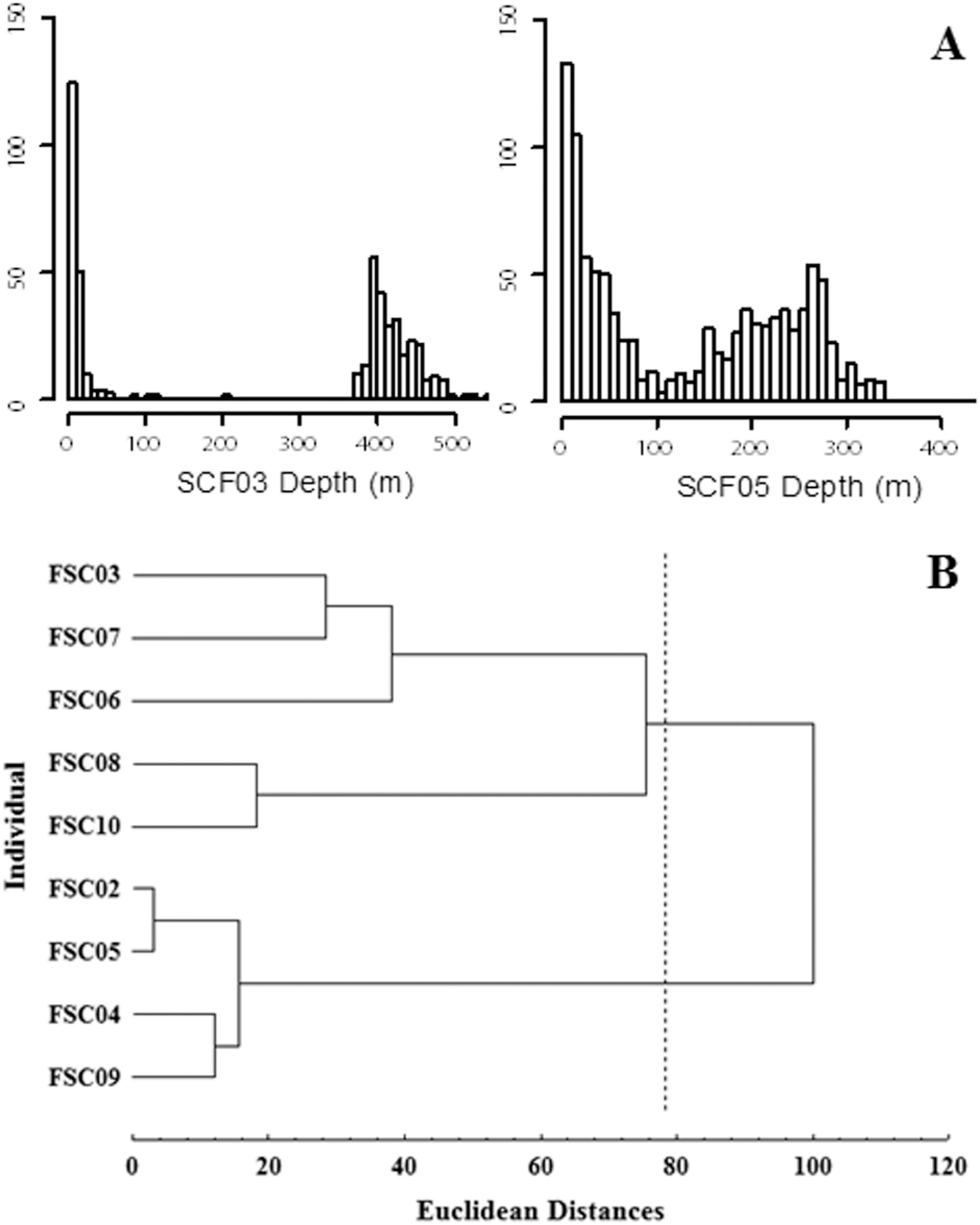
A) Galapagos sea lion females dive depth histograms: SCF03 representing animals who exhibited trips to the north, and SCF05 representing those who directed their trips to the west of the breeding rookery. B) Diving behavior cluster analysis of Galapagos sea lion females. The line indicates the Euclidean distance chosen to define two groups.

The two groups of females showed a bimodal distribution of dive depths, ranging from 20–30 m and from 400–430 m (Fig 4A). A similar but less pronounced pattern was observed in the females that foraged to the west, with dive depths ranging from 20–40 m and 240–350 m (Fig 4A). The diving behavior cluster analysis, based on the deeper dives (>100 m), identified two different groups (Fig 4B). These results are consistent with the spatial segregation observed in their GPS positions. Females from group 1 (deeper dives: SCF03, SCF06, SCF07, SCF08 and SCF10) travelled to the north of the island, characterized by a 600-meter isobath (Fig 3), and the females in group 2 (shallower dives: SCF02, SCF04, SCF05 and SCF09) directed their foraging trips to the west, an area with a 300-meter isobath (Fig 3). We did not obtain location data from SCF10, but her diving behavior (cluster analysis) is consistent with foraging north of the island.

### Stable isotopes analysis

**Fig 5:**
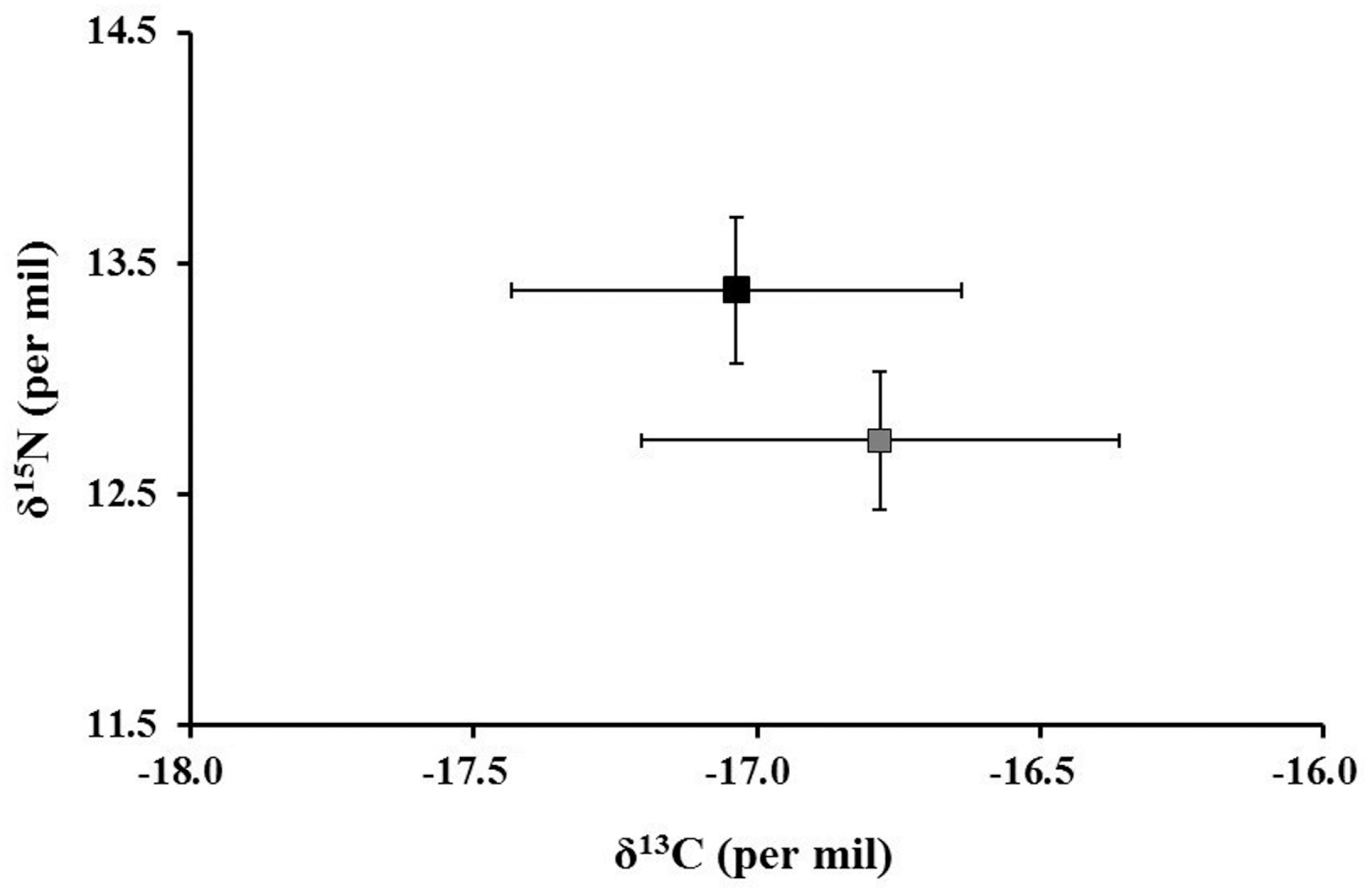
δ^15^N and δ^13^C values (mean ± SD, in %) of Galapagos sea lion females from El Malecón rookery. The black square represents females that travelled north, and grey square represents females that travelled west of the breeding rookery.

Hair samples from the tagged animals were classified into two groups, according to their movement patterns (N = 9, five to the north and four to the west). Mean δ^13^C values were -17.04‰ and -16-78‰ for the females foraging north and west respectively. Mean δ^15^N values were 13.38‰ and 12.73‰ for females that travelled north and west respectively. While no significant differences were observed in the δ^13^C values between the female groups (Paired t test: t_value_ = 0.97, P = 0.356), their δ^15^N values were significantly different (Paired t test: t_value_ = -3.31, p = 0.010) (Fig 5). This suggests trophic level or foraging behavior differences in terms of the prey consumed.

The trophic level estimated for the females foraging north and west was 4.5 and 4.1 respectively. Although both groups are considered tertiary carnivorous consumers, females north are at a slightly higher trophic level than females foraging west.

## Discussion

Populations exposed to a high degree of competition for resource use and habitat occupation exhibit adaptations such as plasticity in their foraging habits, to warrant their survival [11,16]. In this study, *Z*. *wollebaeki* showed a clear intra-population distribution of food resources. This adaptation has contributed to diet diversification and trophic specialization to some degree, facilitating the species survival in an environment deemed demanding for this type of predator [25,45].

Individual distribution across trophic niches within a population could be advantageous for those populations occupying marginal or extreme environments because it reduces competition, facilitating predator survival [3,21]. Because of the otariids of the Galapagos Islands inhabit an ecosystem with fluctuating marine productivity which is strongly influenced by ocean currents and an upwelling pattern [27,28]; these species are continuously struggling to survive in an ecosystem with fluctuating marine productivity associated with oceanographic events characteristic of the region [25,26]. This battle to survive is reflected in their overall reduced and high variation in population size, which is magnified by anomalous oceanographic events, such as El Niño Southern Oscillation (ENSO), which increases mortality [46,47].

### Trophic structure and diet

The diet of *Z*. *wollebaeki* is characterized by a wide trophic spectrum, comprising prey from different environments, including fish families such as: Clupeidae, Myctophidae and Serranidae [24,48]. Despite this great variability, a degree of trophic specialization has been identified in their diet, as a result of the high frequency consumption of a limited number of prey items. The prey species in the *Z*. *wollebaeki* diet, previously identified [3] are consistent with the species found in this study.

The “Galapagos sardine” (*Opisthonema berlangai*) accounted for 31.4% of the *Z*. *wollebaeki* diet. This clupeid feeds mainly on plankton and is abundant in epipelagic areas (0–200 m) with high primary productivity, where forms large schools in shallow waters close to the coast [48,49]; therefore, it is a common prey in the diet of sea lions from the genus *Zalophus* [50,51]. These ecological characteristics make the Galapagos sardine the potential prey of the group of females foraging west of the island, since these animals presented shallower dives and a low trophic level.

On the other hand, the *Z*. *wollebaeki* populations in the south-eastern region of the Archipelago are known to feed on larger fish that provide them with a higher caloric content, such as “camotillos” (*Paralabrax albomaculatus*) or other benthic fish of the Serranidae and Scorpenidae families [24,52]. These fish are mainly carnivorous and are found in deep areas (> 200m) with rocky substrates near the islands [53]. Our results are consistent with previous findings given that *P*. *albomaculatus* accounted for 34.2% of the *Z*. *wollebaeki* diet, becoming the potential prey of the group of females foraging north of the island, since these animals presented deeper dives and a slightly higher trophic level than the other group of females. Based on the PCA results from the scat analysis, we found three main diet types in *Z*. *wollebaeki*, which are contained within foraging strategies epipelagic and benthic. Therefore, the number of diets found in a population does not always reflect the number of foraging strategies.

The significant differences in δ^15^N isotopic values found in the females foraging west and north of the island confirm the two feeding strategies based on the consumption of prey from different trophic levels (herbivorous and carnivorous fish). Our isotopic results agree with the trophic spectrum observed in the diet and the diving behavior, in which the group of females foraging west were represented by a herbivorous fish that lives in the epipelagic zone (0–200 m), such as the *O*. *berlangai* [51], while the group foraging north were represented by a carnivorous fish that lives in deep zone (>200 m), such as the *P*. *albomaculatus* [53]. Although there is an isotopic enrichment in the δ^13^C signatures along the water column, that is related to the highest carbon concentration in benthic algae [38,40], the δ^13^C values were not significantly different between females foraging at the different locations. This could be the result of both groups exhibiting a combination of shallow dives near the coast (possibly by thermoregulation) [45], and deep dives in the range of 100–400 m in their foraging behavior.

### Diving behavior

Previous foraging ecology studies did not consider individual foraging strategies to be common among marine predators or how these would influence their movement and diving patterns, causing intra-population variation [36,54]. However, recent work is beginning to identify the importance of diverse feeding strategies that enable the exploitation of different habitats to reduce competition and thus facilitate survival [55,56,57].

The variation in foraging patterns (pelagic/benthic) within a population has been associated with differences in body size or the biogeographic region where they live [1,22,58]. Australian (*Neophoca cinerea*) and South American sea lions (*Otaria flavescens*), are considered benthic foragers due to the extent and depth of the continental shelf around their rookeries [21,59,60]. California (*Zalophus californianus*) and New Zealand (*Phocarctos hookeri*) sea lions, show a preference for epipelagic areas, where the continental shelf is deeper [61,62,63]. The *Z*. *wollebaeki* females in the central region of the Archipelago exhibit three foraging strategies (epipelagic, mesopelagic and benthic) [31]. This behavior creates a distribution of the trophic niche to reduce competition among females, which is intensified by the extended nursing period and pup care that prevails in this species [64,65].

In this work, we identified two of the strategies reported for the species (epipelagic/benthic) [31]. Such strategies were related to the location of their foraging areas and the bathymetric profile of each site. For example, females foraging west (isobaths -200) showed an epipelagic strategy represented by shallower dives, due to the seek and capture of epipelagic fish such as the "Galapagos sardines" (*O*. *berlangai*). While females foraging north (isobaths -600) showed a benthic strategy associated to deep dives, directed to consume fish of deep zones such as the "camotillos" (*P*. *albomaculatus*). This demonstrates that the number of foraging strategies exhibited by the species varies with colony, and is likely associated to prey availability and oceanographic characteristics of the area.

### Intraspecific variation

As previously reported by Páez-Rosas & Aurioles-Gamboa 2010 and Villegas-Amtmann et al. 2008, we expected that sea lions from San Cristobal would also exhibit a foraging niche distribution. We found variation in their diet and δ^15^N isotopic values (a trophic level proxy), together with spatial differences in their foraging trips and diving behavior, providing further evidence that the individuals of this species utilize specific foraging niches in order to decrease competition for resources [14]. This niche expansion, or 'ecological release', has been widely described in other insular marine predators [49,60], and it is associated to variability in marine productivity and the availability of 'open' niches due to the absence of predators [66].

The utilization of specific feeding areas, together with a higher and more frequent consumption of certain prey, reduces competition within a population [14,17]. However, individual preferences must have certain flexibility to incorporate other prey in their diets, which might prove useful when competition intensifies or resource abundance fluctuates [66]. Therefore, although the diets of different groups of individuals are characterized by a few 'important' prey species, they should be diverse and share certain groups of prey. This would allow individuals to accommodate to environmental changes when resources are scarce by sharing diet preferences [7]. The *Z*. *wollebaeki* trophic spectrum provides a good example for this, because although its diet is characterized by two main prey items, it also includes a group of four or five prey with a high index of importance.

In summary, by applying an array of different techniques utilized in foraging ecology studies, our study provides evidence of intrinsic variation in the foraging behavior of *Z*. *wollebaeki*. We identified diet variability as a result of a differential usage of foraging areas. Based on the regional differences found in the trophic behavior of this species [24,65], further studies should be done to consider other rookeries with contrasting marine productivity and population number (e.g., the western and north regions of the Archipelago), which directly affect the degree of competition within a population.

## Supporting information

S1 File Database of foraging trip variables and tracking Galapagos sea lions. (XLS)

S2 File Database of diving behavior and tracking Galapagos sea lions. (XLS)

S3 File Isotopic data of Galapagos sea lions. (XLS)
